# In Memoriam-Professor Ajit K.Padhy

**Published:** 2013

**Authors:** Emerita Andres Barrenechea


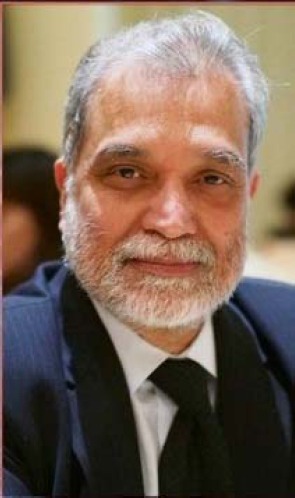


Dr. Ajit Kumar Padhy MD, FAMS was a Senior Consultant, Department of Nuclear Medicine & PET at Singapore General Hospital, Singapore when he passed away on 22^nd^ August 2013. Prior to this, he was Head of the Nuclear Medicine section International Atomic Energy Agency (IAEA), in Vienna for seven years. He was also Head of Nuclear Medicine at the All India Institute of Nuclear medicine, New Delhi, India.

His untimely demise left a wife and two loving sons, accomplished in their own rights.

In the realm of Nuclear Medicine, the specialty he loved so much, he left a worldwide league of physicians and colleagues orphaned. His passion of the specialty will remain unsurpassed.

His selfless devotion to uplift the practice of Nuclear Medicine in developing countries, encourage young doctors to do research and his peers to excel in this field was limitless.

Born in Orissa, where he enjoyed his childhood, he will remain a treasure that belongs to the entire world. He loved life, and was even described by his classmates to be “enterprising and flamboyant”. He loved music, movies and dancing. He also loves to cook and his cooking was delicious. To us his colleagues, he was stylish and paid attention to small details when it comes to arranging meetings/conferences (being in the IAEA for 7 years and doing this around the world). He is so confident, kind and generous. He was honest and critical especially if it will be for your betterment and without intention to hurt.

In 2009, he co-founded the World Association of Radiopharmaceutical and Molecular Therapy where he was President till 2012 when he became the Executive Director. WARMTH now has more than 400 members around the globe and his loss is so strongly felt. He is also Editor in Chief of the “World Journal of Nuclear Medicine” at the time of his death. This was another “baby” of his, spending sleepless nights beating the deadline. He was also an active member of editorial board of the “Asia Oceania Journal of Nuclear Medicine & biology”. He had hundreds of published papers and chapter of books. His advocacy for therapy for thyroid cancer, thyrotoxicosis and liver cancer are seen in most of his works.

To all of us whose lives he has touched, we deeply mourn his passing away.

His challenge to make a better world through therapy especially in oncology must be a reminder to those he left behind.

We must continue the passion and keep the fire burning for his works, a legacy he left. He will always be an inspiration for us. May he attain peace, and happiness in the life beyond.

